# Ethane-1,2-diaminium 2,2′-[tereph­thal­oyl­bis(aza­nedi­yl)]di­acetate tetrahydrate

**DOI:** 10.1107/S1600536813029632

**Published:** 2013-11-06

**Authors:** Niels-Patrick Pook, Mimoza Gjikaj, Arnold Adam

**Affiliations:** a Institute of Inorganic and Analytical Chemistry, Clausthal University of Technology, Paul-Ernst-Str. 4, D-38678, Clausthal-Zellerfeld, Germany

## Abstract

In the title salt hydrate, C_2_H_10_N_2_^2+^·C_12_H_10_N_2_O_6_^2−^·4H_2_O, each of the ions is located about a centre of inversion and the asymmetric unit is completed by two water molecules in general positons. In the crystal, the cations, anions and water mol­ecules are connected by O—H⋯O and N—H⋯O hydrogen bonding into a three-dimensional network.

## Related literature

The starting material, 2,2′-(benzene-1,4-dicarboxamido)­diacetatic acid, was prepared by the method of Cleaver & Pratt (1955 ▶). For related organic structures, see: Armelin *et al.* (2001 ▶); Ray *et al.* (2006 ▶). For crystal strucutres of *d*-block elements with 2,2′-(terephthaloylbis(aza­nedi­yl))di­acetate and similar ligands, see: Duan *et al.* (2010 ▶); Kostakis *et al.* (2005 ▶, 2011 ▶); Wisser *et al.* (2008 ▶); Zhang & You (2005) ▶; Zhang *et al.* (2006 ▶). 
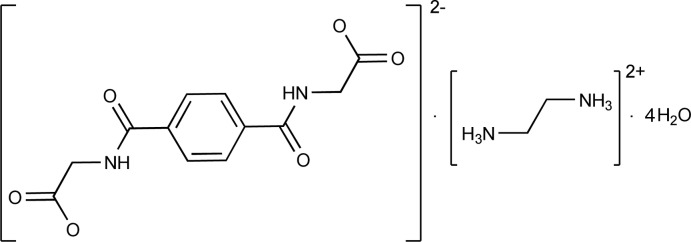


## Experimental

### 

#### Crystal data

C_2_H_10_N_2_^2+^·C_12_H_10_N_2_O_6_^2−^·4H_2_O
*M* *_r_* = 412.40
Monoclinic, 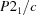

*a* = 7.3710 (11) Å
*b* = 9.0675 (11) Å
*c* = 14.704 (2) Å
β = 105.041 (11)°
*V* = 949.1 (2) Å^3^
*Z* = 2
Mo *K*α radiation
μ = 0.12 mm^−1^
*T* = 223 K
0.28 × 0.25 × 0.22 mm

#### Data collection

Stoe IPDS 2 diffractometer
9883 measured reflections
1859 independent reflections
1633 reflections with *I* > 2σ(*I*)
*R*_int_ = 0.099

#### Refinement

*R*[*F* ^2^ > 2σ(*F* ^2^)] = 0.038
*wR*(*F* ^2^) = 0.096
*S* = 1.09
1859 reflections
183 parameters
All H-atom parameters refined
Δρ_max_ = 0.27 e Å^−3^
Δρ_min_ = −0.27 e Å^−3^

### 

Data collection: *X-AREA* (Stoe, 2008 ▶); cell refinement: *X-AREA*; data reduction: *X-AREA*; program(s) used to solve structure: *SHELXS97* (Sheldrick, 2008 ▶); program(s) used to refine structure: *SHELXL97* (Sheldrick, 2008 ▶); molecular graphics: *ORTEP-3 for Windows* (Farrugia 2012 ▶); software used to prepare material for publication: *SHELXL97*, *PLATON* (Spek, 2009 ▶) and *publCIF* (Westrip, 2010 ▶).

## Supplementary Material

Crystal structure: contains datablock(s) I. DOI: 10.1107/S1600536813029632/nc2319sup1.cif

Structure factors: contains datablock(s) I. DOI: 10.1107/S1600536813029632/nc2319Isup2.hkl

Supplementary material file. DOI: 10.1107/S1600536813029632/nc2319Isup3.cdx

Supplementary material file. DOI: 10.1107/S1600536813029632/nc2319Isup4.cml

Additional supplementary materials:  crystallographic information; 3D view; checkCIF report

## Figures and Tables

**Table 1:** Hydrogen-bond geometry (Å, °)

*D*—H⋯*A*	*D*—H	H⋯*A*	*D*⋯*A*	*D*—H⋯*A*
O4—H4*B*⋯O3	0.89 (3)	1.80 (3)	2.6792 (16)	169 (2)
N2—H2*B*⋯O3	0.96 (2)	1.85 (2)	2.7896 (17)	164.3 (18)
O4—H4*A*⋯O1^i^	0.87 (3)	2.03 (3)	2.8741 (16)	162 (2)
O5—H5*A*⋯O2^ii^	0.92 (3)	1.87 (3)	2.7669 (17)	168 (2)
O5—H5*B*⋯O1^iii^	0.86 (3)	2.01 (3)	2.8641 (17)	169 (3)
N1—H1⋯O4^iv^	0.87 (2)	2.02 (2)	2.8509 (17)	159.8 (18)
N2—H2*A*⋯O5^v^	0.93 (2)	1.91 (2)	2.8278 (18)	172.6 (18)
N2—H2*C*⋯O2^vi^	0.93 (2)	1.89 (2)	2.8086 (17)	169 (2)

Symmetry codes: (i) 
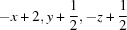
; (ii) 
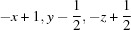
; (iii) 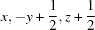; (iv) 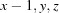; (v) 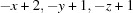; (vi) 
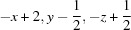
.

**Fig. 1.**
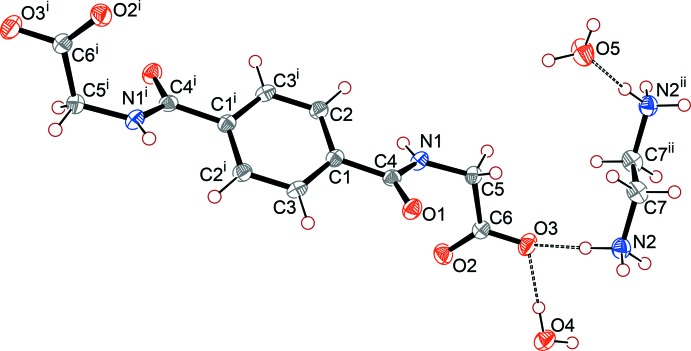
View of the asymmetric unit of the title compound with labeling and displacement ellipsoids drawn at the 50% probability level. The dashed lines indicate N—H···O and O—H···O hydrogen bonds (see Table 1 for details). Symmetry codes: (i) -x - 1, -y - 1, -1 - z; (ii) -x + 1, -y - 1, -z.

**Fig. 2.**
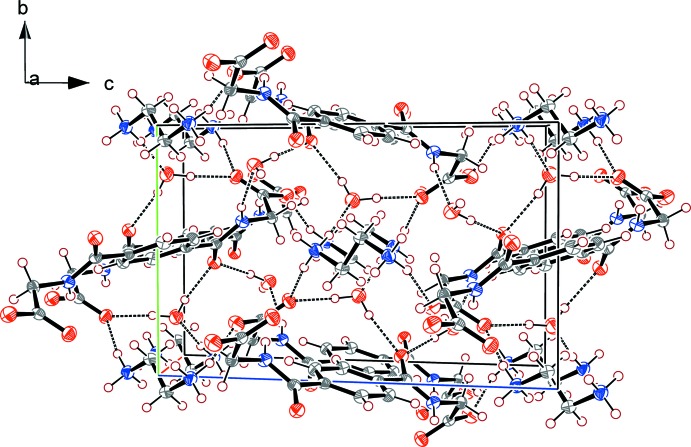
Crystal structure of the title compound viewed along the a axis. Displacement ellipsoids are drawn at the 50% probability level and hydrogen bonding is shown as dashed lines. Hydrogen atoms not involved in hydrogen bonding have been omitted for clarity.

## supplementary crystallographic information

### 1. Comment

Recently, the inter­est in constructing metal-organic coordination polymers continuously rises due to their facinating structures and properties with potential application as functional materials. For the synthesis of such compounds frequently relatively rigid ligands with several coordination centers are used. In this context the title compound was prepared, which should be used as a precursor in the synthesis of new coordination polymers.

The crystal structure of the title compound consists of 2,2'-(terephthaloylbis(aza­nediyl))di­acetate anions that are located on centers of inversion as well as of ethyl­enediaminium cations and water molecules in general positions (Fig. 1). The ethyl­enedi­amine cations are connected to the anions by N—H···O hydrogen bonding between the carboxyl­ate O atom and the ammonium H atoms. The anions and cations are additionally linked to water molecules via N—H···O and O—H···O hydrogen bonding into a three-dimensional network (Fig. 2 and Table 1). Furthermore, the compound shows luminescence under excitation with ultraviolet light.

### 2. Experimental

The starting material, 2,2'-(benzene-1,4-dicarboxamido)­diacetatic acid, was prepared by the method of Cleaver & Pratt (Cleaver & Pratt, 1955). Single crystals were obtained by slow evaporation from an aqueous solution (40 ml) of 2,2'-terephthaloylbis-(glycine) dihydrate (630 mg) and ethyl­enedi­amine (3 ml) at room temperature. Colorless block shaped crystals appeared after a few days.

### 2.1. Refinement

All hydrogen atoms were located the difference Fourier map and were refined isotropically with no restraints.

### Crystal data

**Table:**

C_2_H_10_N_2_^2+^·C_12_H_10_N_2_O_6_^2^^−^·4H_2_O	*F*(000) = 440
*M**_r_* = 412.40	*D*_x_ = 1.443 Mg m^−^^3^
Monoclinic, *P*2_1_/*c*	Mo *K*α radiation, λ = 0.71073 Å
Hall symbol: -P 2ybc	Cell parameters from 1859 reflections
*a* = 7.3710 (11) Å	θ = 1.0–26.0°
*b* = 9.0675 (11) Å	µ = 0.12 mm^−^^1^
*c* = 14.704 (2) Å	*T* = 223 K
β = 105.041 (11)°	Block, colorless
*V* = 949.1 (2) Å^3^	0.28 × 0.25 × 0.22 mm
*Z* = 2	

### Data collection

**Table:**

Stoe IPDS 2 diffractometer	1633 reflections with *I* > 2σ(*I*)
Radiation source: fine-focus sealed tube	*R*_int_ = 0.099
Graphite monochromator	θ_max_ = 26.0°, θ_min_ = 2.7°
ω–scans	*h* = −9→9
9883 measured reflections	*k* = −11→11
1859 independent reflections	*l* = −18→18

### Refinement

**Table:**

Refinement on *F*^2^	Primary atom site location: structure-invariant direct methods
Least-squares matrix: full	Secondary atom site location: difference Fourier map
*R*[*F*^2^ > 2σ(*F*^2^)] = 0.038	Hydrogen site location: inferred from neighbouring sites
*wR*(*F*^2^) = 0.096	All H-atom parameters refined
*S* = 1.09	*w* = 1/[σ^2^(*F*_o_^2^) + (0.0383*P*)^2^ + 0.2529*P*] where *P* = (*F*_o_^2^ + 2*F*_c_^2^)/3
1859 reflections	(Δ/σ)_max_ < 0.001
183 parameters	Δρ_max_ = 0.27 e Å^−^^3^
0 restraints	Δρ_min_ = −0.27 e Å^−^^3^

### Special details

**Table:**

Geometry. All e.s.d.'s (except the e.s.d. in the dihedral angle between two l.s. planes) are estimated using the full covariance matrix. The cell e.s.d.'s are taken into account individually in the estimation of e.s.d.'s in distances, angles and torsion angles; correlations between e.s.d.'s in cell parameters are only used when they are defined by crystal symmetry. An approximate (isotropic) treatment of cell e.s.d.'s is used for estimating e.s.d.'s involving l.s. planes.
Refinement. Refinement of *F*^2^ against ALL reflections. The weighted *R*-factor *wR* and goodness of fit *S* are based on *F*^2^, conventional *R*-factors *R* are based on *F*, with *F* set to zero for negative *F*^2^. The threshold expression of *F*^2^ > σ(*F*^2^) is used only for calculating *R*-factors(gt) *etc*. and is not relevant to the choice of reflections for refinement. *R*-factors based on *F*^2^ are statistically about twice as large as those based on *F*, and *R*- factors based on ALL data will be even larger.

### Fractional atomic coordinates and isotropic or equivalent isotropic displacement parameters (Å^2^)

**Table:**

	*x*	*y*	*z*	*U*_iso_*/*U*_eq_	
O1	0.49836 (13)	0.43561 (11)	0.11941 (7)	0.0248 (2)	
O2	0.67537 (15)	0.79044 (11)	0.17064 (7)	0.0293 (3)	
O3	0.87997 (14)	0.70796 (12)	0.30002 (8)	0.0307 (3)	
O4	1.16417 (16)	0.83739 (12)	0.24811 (9)	0.0304 (3)	
H4A	1.250 (3)	0.875 (3)	0.2948 (17)	0.052 (6)*	
H4B	1.067 (3)	0.806 (3)	0.2678 (16)	0.050 (6)*	
O5	0.57247 (18)	0.30272 (13)	0.50636 (9)	0.0387 (3)	
H5A	0.505 (3)	0.294 (3)	0.4449 (19)	0.058 (7)*	
H5B	0.539 (4)	0.227 (3)	0.534 (2)	0.073 (8)*	
N1	0.39210 (16)	0.60948 (13)	0.20250 (8)	0.0216 (3)	
H1	0.302 (3)	0.670 (2)	0.2048 (14)	0.037 (5)*	
N2	1.13211 (18)	0.50461 (14)	0.40607 (9)	0.0256 (3)	
H2A	1.234 (3)	0.561 (2)	0.4374 (14)	0.035 (5)*	
H2B	1.039 (3)	0.560 (2)	0.3615 (15)	0.041 (5)*	
H2C	1.183 (3)	0.434 (3)	0.3741 (16)	0.053 (6)*	
C1	0.17640 (18)	0.50869 (14)	0.06420 (9)	0.0192 (3)	
C2	0.01507 (19)	0.54693 (15)	0.09178 (10)	0.0218 (3)	
H2	0.023 (2)	0.5766 (19)	0.1573 (13)	0.028 (4)*	
C3	0.16018 (19)	0.46137 (15)	−0.02754 (10)	0.0220 (3)	
H3	0.268 (2)	0.4371 (18)	−0.0461 (12)	0.021 (4)*	
C4	0.36832 (18)	0.51552 (14)	0.13090 (9)	0.0194 (3)	
C5	0.56927 (18)	0.62239 (15)	0.27321 (10)	0.0215 (3)	
H51	0.625 (2)	0.527 (2)	0.2943 (12)	0.025 (4)*	
H52	0.544 (3)	0.673 (2)	0.3279 (14)	0.033 (5)*	
C6	0.71953 (19)	0.71403 (14)	0.24383 (10)	0.0205 (3)	
C7	1.0404 (2)	0.43916 (16)	0.47572 (11)	0.0275 (3)	
H7A	1.135 (3)	0.384 (2)	0.5210 (14)	0.036 (5)*	
H7B	0.937 (3)	0.375 (2)	0.4412 (13)	0.033 (5)*	

### Atomic displacement parameters (Å^2^)

**Table:**

	*U*^11^	*U*^22^	*U*^33^	*U*^12^	*U*^13^	*U*^23^
O1	0.0196 (5)	0.0265 (5)	0.0275 (5)	0.0040 (4)	0.0048 (4)	−0.0023 (4)
O2	0.0294 (6)	0.0305 (5)	0.0258 (6)	−0.0051 (4)	0.0034 (4)	0.0053 (4)
O3	0.0208 (5)	0.0308 (6)	0.0363 (6)	−0.0048 (4)	−0.0001 (4)	0.0053 (4)
O4	0.0237 (6)	0.0322 (6)	0.0349 (6)	−0.0038 (4)	0.0070 (5)	−0.0053 (5)
O5	0.0466 (7)	0.0325 (6)	0.0307 (7)	−0.0133 (5)	−0.0010 (5)	0.0009 (5)
N1	0.0157 (5)	0.0219 (6)	0.0266 (6)	0.0004 (4)	0.0041 (5)	−0.0026 (4)
N2	0.0255 (6)	0.0263 (6)	0.0237 (6)	0.0008 (5)	0.0040 (5)	−0.0018 (5)
C1	0.0180 (6)	0.0154 (6)	0.0237 (7)	−0.0017 (5)	0.0047 (5)	0.0024 (5)
C2	0.0204 (7)	0.0224 (7)	0.0230 (7)	−0.0017 (5)	0.0065 (5)	−0.0016 (5)
C3	0.0170 (6)	0.0226 (7)	0.0281 (7)	−0.0007 (5)	0.0087 (5)	−0.0016 (5)
C4	0.0189 (6)	0.0177 (6)	0.0227 (7)	−0.0011 (5)	0.0070 (5)	0.0033 (5)
C5	0.0201 (7)	0.0229 (7)	0.0204 (7)	−0.0011 (5)	0.0033 (5)	−0.0010 (6)
C6	0.0202 (7)	0.0181 (6)	0.0228 (7)	−0.0006 (5)	0.0050 (5)	−0.0038 (5)
C7	0.0358 (8)	0.0206 (7)	0.0260 (8)	0.0025 (6)	0.0076 (6)	−0.0010 (6)

### Geometric parameters (Å, º)

**Table:**

O1—C4	1.2476 (16)	C1—C3	1.391 (2)
O2—C6	1.2500 (18)	C1—C2	1.3961 (19)
O3—C6	1.2558 (17)	C1—C4	1.4990 (18)
O4—H4A	0.87 (3)	C2—C3^i^	1.390 (2)
O4—H4B	0.89 (3)	C2—H2	0.987 (19)
O5—H5A	0.92 (3)	C3—C2^i^	1.390 (2)
O5—H5B	0.86 (3)	C3—H3	0.931 (17)
N1—C4	1.3301 (18)	C5—C6	1.5337 (18)
N1—C5	1.4476 (17)	C5—H51	0.977 (18)
N1—H1	0.87 (2)	C5—H52	0.98 (2)
N2—C7	1.489 (2)	C6—O3	1.2558 (17)
N2—H2A	0.93 (2)	C7—C7^ii^	1.517 (3)
N2—H2B	0.96 (2)	C7—H7A	0.97 (2)
N2—H2C	0.93 (2)	C7—H7B	0.98 (2)
			
H4A—O4—H4B	110 (2)	O1—C4—N1	122.09 (12)
H5A—O5—H5B	104 (2)	O1—C4—C1	121.01 (12)
C4—N1—C5	121.96 (12)	N1—C4—C1	116.90 (11)
C4—N1—H1	119.3 (13)	N1—C5—C6	115.11 (12)
C5—N1—H1	118.6 (13)	N1—C5—H51	112.5 (10)
C7—N2—H2A	109.4 (12)	C6—C5—H51	107.3 (10)
C7—N2—H2B	108.1 (12)	N1—C5—H52	107.5 (11)
H2A—N2—H2B	113.2 (17)	C6—C5—H52	106.3 (11)
C7—N2—H2C	112.9 (14)	H51—C5—H52	107.8 (15)
H2A—N2—H2C	104.4 (18)	O2—C6—O3	125.53 (13)
H2B—N2—H2C	108.9 (18)	O2—C6—O3	125.53 (13)
C3—C1—C2	119.54 (13)	O2—C6—C5	119.81 (12)
C3—C1—C4	118.45 (12)	O3—C6—C5	114.62 (12)
C2—C1—C4	122.01 (13)	O3—C6—C5	114.62 (12)
C3^i^—C2—C1	120.03 (14)	N2—C7—C7^ii^	109.72 (15)
C3^i^—C2—H2	119.1 (10)	N2—C7—H7A	107.8 (12)
C1—C2—H2	120.9 (10)	C7^ii^—C7—H7A	110.7 (12)
C2^i^—C3—C1	120.43 (13)	N2—C7—H7B	107.9 (11)
C2^i^—C3—H3	120.1 (10)	C7^ii^—C7—H7B	109.0 (11)
C1—C3—H3	119.5 (10)	H7A—C7—H7B	111.6 (16)
			
C3—C1—C2—C3^i^	−0.5 (2)	C3—C1—C4—N1	154.93 (12)
C4—C1—C2—C3^i^	−179.87 (12)	C2—C1—C4—N1	−25.66 (18)
C2—C1—C3—C2^i^	0.5 (2)	C4—N1—C5—C6	79.20 (16)
C4—C1—C3—C2^i^	179.89 (12)	O3—O3—C6—O2	0.00 (17)
C5—N1—C4—O1	−1.6 (2)	O3—O3—C6—C5	0.00 (13)
C5—N1—C4—C1	177.93 (11)	N1—C5—C6—O2	11.70 (18)
C3—C1—C4—O1	−25.48 (18)	N1—C5—C6—O3	−170.64 (12)
C2—C1—C4—O1	153.92 (13)	N1—C5—C6—O3	−170.64 (12)

Symmetry codes: (i) −*x*, −*y*+1, −*z*; (ii) −*x*+2, −*y*+1, −*z*+1.

### Hydrogen-bond geometry (Å, º)

**Table:**

*D*—H···*A*	*D*—H	H···*A*	*D*···*A*	*D*—H···*A*
O4—H4*B*···O3	0.89 (3)	1.80 (3)	2.6792 (16)	169 (2)
N2—H2*B*···O3	0.96 (2)	1.85 (2)	2.7896 (17)	164.3 (18)
O4—H4*A*···O1^iii^	0.87 (3)	2.03 (3)	2.8741 (16)	162 (2)
O5—H5*A*···O2^iv^	0.92 (3)	1.87 (3)	2.7669 (17)	168 (2)
O5—H5*B*···O1^v^	0.86 (3)	2.01 (3)	2.8641 (17)	169 (3)
N1—H1···O4^vi^	0.87 (2)	2.02 (2)	2.8509 (17)	159.8 (18)
N2—H2*A*···O5^ii^	0.93 (2)	1.91 (2)	2.8278 (18)	172.6 (18)
N2—H2*C*···O2^vii^	0.93 (2)	1.89 (2)	2.8086 (17)	169 (2)

Symmetry codes: (ii) −*x*+2, −*y*+1, −*z*+1; (iii) −*x*+2, *y*+1/2, −*z*+1/2; (iv) −*x*+1, *y*−1/2, −*z*+1/2; (v) *x*, −*y*+1/2, *z*+1/2; (vi) *x*−1, *y*, *z*; (vii) −*x*+2, *y*−1/2, −*z*+1/2.
